# Altered Insular and Occipital Responses to Simulated Vertical Self-Motion in Patients with Persistent Postural-Perceptual Dizziness

**DOI:** 10.3389/fneur.2017.00529

**Published:** 2017-10-17

**Authors:** Roberta Riccelli, Luca Passamonti, Nicola Toschi, Salvatore Nigro, Giuseppe Chiarella, Claudio Petrolo, Francesco Lacquaniti, Jeffrey P. Staab, Iole Indovina

**Affiliations:** ^1^Laboratory of Neuromotor Physiology, IRCCS Santa Lucia Foundation, Rome, Italy; ^2^Centre of Space BioMedicine, University of Rome Tor Vergata, Rome, Italy; ^3^Department of Clinical Neurosciences, University of Cambridge, Cambridge, United Kingdom; ^4^Institute of Bioimaging and Molecular Physiology, National Research Council, Catanzaro, Italy; ^5^Department of Biomedicine and Prevention, University of Rome Tor Vergata, Rome, Italy; ^6^Department of Radiology, Athinoula A. Martinos Center for Biomedical Imaging, Boston, MA, United States; ^7^Unit of Audiology, Department of experimental and clinical medicine, Magna Græcia University, Catanzaro, Italy; ^8^Department of Psychiatry and Psychology, Mayo Clinic, Rochester, MN, United States; ^9^Department of Otorhinolaryngology – Head and Neck Surgery, Mayo Clinic, Rochester, MN, United States

**Keywords:** rollercoaster simulation, vestibular system, functional magnetic resonance imaging, persistent postural-perceptual dizziness, chronic subjective dizziness, insula

## Abstract

**Background:**

Persistent postural-perceptual dizziness (PPPD) is a common functional vestibular disorder characterized by persistent symptoms of non-vertiginous dizziness and unsteadiness that are exacerbated by upright posture, self-motion, and exposure to complex or moving visual stimuli. Recent physiologic and neuroimaging data suggest that greater reliance on visual cues for postural control (as opposed to vestibular cues—a phenomenon termed visual dependence) and dysfunction in central visuo-vestibular networks may be important pathophysiologic mechanisms underlying PPPD. Dysfunctions are thought to involve insular regions that encode recognition of the visual effects of motion in the gravitational field.

**Methods:**

We tested for altered activity in vestibular and visual cortices during self-motion simulation obtained *via* a visual virtual-reality rollercoaster stimulation using functional magnetic resonance imaging in 15 patients with PPPD and 15 healthy controls (HCs). We compared between groups differences in brain responses to simulated displacements in vertical vs horizontal directions and correlated the difference in directional responses with dizziness handicap in patients with PPPD.

**Results:**

HCs showed increased activity in the anterior bank of the central insular sulcus during vertical relative to horizontal motion, which was not seen in patients with PPPD. However, for the same comparison, dizziness handicap correlated positively with activity in the visual cortex (V1, V2, and V3) in patients with PPPD.

**Conclusion:**

We provide novel insight into the pathophysiologic mechanisms underlying PPPD, including functional alterations in brain processes that affect balance control and reweighting of space-motion inputs to favor visual cues. For patients with PPPD, difficulties using visual data to discern the effects of gravity on self-motion may adversely affect balance control, particularly for individuals who simultaneously rely too heavily on visual stimuli. In addition, increased activity in the visual cortex, which correlated with severity of dizziness handicap, may be a neural correlate of visual dependence.

## Introduction

Persistent postural-perceptual dizziness (PPPD) is a chronic functional vestibular disorder that manifests with dizziness, unsteadiness, and swaying or rocking (non-spinning) vertigo that wax and wane throughout the day. These symptoms are exacerbated by upright posture, active or passive self-motion, and exposure to environments with complex or moving visual stimuli (1–3). The definition of PPPD (2) was derived from four precursors that have been described in the neuro-otologic literature over the last 30 years, namely, phobic postural vertigo (PPV) (4), space-motion discomfort (5), visual vertigo (6), and chronic subjective dizziness (CSD) (7).

The most common triggers of PPPD are peripheral vestibular conditions such as vestibular neuritis (VN) and benign paroxysmal positional vertigo (BPPV), although vestibular migraine, central vestibular disorders, and non-vestibular conditions such as panic attacks, mild traumatic brain injuries, and orthostatic intolerance have also been reported as precipitants of PPPD (8) or its precursors (4, 6, 9, 10). Cross-sectional investigations of patients with PPV (11, 12) and CSD (13) as well as prospective studies that followed patients with acute VN from the onset of illness for at least 6 months thereafter (14–16) identified two physiological changes that may be applicable to PPPD (2). These are (a) utilization of a stiffened postural control strategy (11–13) and (b) greater reliance on visual vs vestibular inputs for balance control, commonly termed visual dependence (14–16).

Healthy people employ these strategies transiently under conditions of balance threat such as standing on an elevated platform (17, 18) or walking across slippery surfaces. Patients with neuro-otologic disorders use them when vestibular or somatosensory inputs are compromised by conditions such as peripheral or central vestibular dysfunction (6) or sensory neuropathies of the feet. Emerging evidence suggests that patients with PPPD continue to manifest stiffened postural control and visual dependence long after the precipitating conditions have remitted (3, 8). It is thought that this unnecessary use of high-risk strategies makes patients with PPPD more vulnerable to increased symptoms and greater disruptions to postural stability when exposed to space and motion stimuli that are encountered in routine daily activities (1). Prospective studies have shown that the development of persistent dizziness is not related to the extent of peripheral vestibular injury when a structural vestibular disorder is the precipitating event, but rather to the emergence of altered postural control (19) and ongoing visual dependence (14).

In this context, functional MRI data of healthy individuals (20, 21), patients with visually induced dizziness (one of the key symptoms of PPPD) (22), and patients with PPPD itself (23) revealed reduced response as well as reduced connectivity in a series of regions belonging to the multimodal vestibular cortex and involved in threat assessment and spatial orientation, namely, the insula, inferior frontal gyrus, hippocampus, anterior cingulate cortex, and superior temporal gyrus.

In another series of experiments, space-motion stimuli simulating self-motion along vertical and horizontal directions were compared in healthy individuals to study the visual effects of motion within the earth’s gravitation field on brain activity (24, 25). Recognition of the visual effects of gravity on body motion facilitates prediction of body motion in space to support balance control (26, 27). Increased activity consistent with recognition of gravity law was found in regions adjacent to the central insular sulcus (25). Altered processing of gravitational motion was found in patients with stroke lesions in perisylvian regions adjacent to the posterior insula (28). Given the adverse effects of upright posture and visual motion stimuli on patients with PPPD, altered activity in these regions may be particularly applicable to pathophysiologic processes underlying this disorder.

While these MRI studies found alterations in brain activity, connectivity and structure in areas responsible for processing vestibular and visual information, all relevant to PPPD, more detailed information is needed to better understand functional alterations in response to space-motion stimuli and how they relate to the clinical symptoms of PPPD.

The aim of this study was to investigate brain activity associated with increased sensitivity to visual motion stimuli in patients with PPPD, and in particular, to assess the state of cortical mechanisms linked to recognition of visual inputs that indicate self-motion in the gravitational field. We hypothesized that one of the causes of dysfunctional postural control in PPPD may be an alteration of this mechanism in the insula. More specifically, consistent with literature on visual dependence and postural control in patients with persistent dizziness (14, 15) and PPPD (8, 15), we expected that patients with PPPD would display decreased brain activity in areas that process vestibular stimuli (particularly the insula) and increased responses in the visual system (primarily occipital areas) when comparing vertical vs horizontal simulated self-motion.

## Materials and Methods

### Participants

Fifteen patients with PPPD and 15 healthy volunteers were included in the analyses. All participants gave written informed consent to participate in the study, which was approved by the University of Catanzaro Research Ethics Committee, according to the Helsinki declaration.^1^ The same individuals also took part in another series of experiments reported in previous studies (20, 21, 23). All participants were right-handed, as assessed *via* the Edinburgh Handedness Inventory (29). To measure personality traits of the five Factor Model (neuroticism, extraversion, openness, agreeableness, and conscientiousness), participants completed a computerized version of the Italian translation of the Revised NEO Personality Inventory (NEO-PI-R) (30). We used the Mini-International Neuropsychiatric Interview (MINI) to identify psychiatric diagnoses (31) and the Generalized Anxiety Disorder questionnaire (GAD-7) (32) and Patient Health Questionnaire (PHQ-9) (33) to assess severity of anxiety and depression, respectively. We also assessed susceptibility of all participants to motion sickness using the Motion Sickness Susceptibility Questionnaire (MSSQ) (34) and the severity of dizziness handicap in patients with PPPD using the Dizziness Handicap Inventory (DHI) (35). None of the participants had histories of migraine or other neurological or psychiatric disorders that required submission to psychiatric care or psychopharmacological treatment.

Diagnostic criteria for PPPD were as follows: (1) persistent non-vertiginous dizziness, unsteadiness, or both, lasting 3 months or more, (2) symptoms present most days, throughout the day (although they may wax and wane in severity), (3) symptoms exacerbated by upright posture, exposure to moving or complex visual stimuli, and active or passive head motion (ICD-11^2^). Exclusion criteria included active neuro-otologic disorders other than PPPD, chronic medical illnesses, pregnancy, medication use, smoking, and a history of migraine or head injury. History of quiescent or fully compensated vestibular peripheral deficits at the time of study was not an exclusion criterion. This was because otologic illnesses are known to be the most common triggers of PPPD (10, 36), as was the case in our patient group. In particular, most of our patients with PPPD had a history of VN (*N* = 12), while a few of them had experienced BPPV (*N* = 2) or both VN and BPPV (*N* = 1). These disturbances were localized on the right side in seven patients, left side in seven patients, or bilaterally in one patient. Patients with PPPD who had VN underwent caloric testing in the acute stage of their peripheral vestibular disease and 6 months later to evaluate the adequacy of their recovery. The percentage of reduced vestibular response on the electronystagmogram was calculated using the Jongkees’ formula (37), which revealed mild to medium unilateral canal paresis (relative vestibular reduction in the nystagmus slow-phase velocity peak) across patients in the acute stage (mean = 35%, range 25–45%) and return to normal values 6 months later (mean = 13%, range 5–20%). Patients who experienced BPPV as a trigger for PPPD had no symptoms or signs of active positional vertigo at the time of entry into the study. The duration of illness for patients with PPPD ranged from 8 to 120 months with a median of 18 months and mean ± SD of 32.5 ± 34.8 months. DHI scores for patients with PPPD ranged from 10 to 60, indicating a range of low to severe handicap with a mean ± SD of 34 ± 16.1 (35). In a confirmatory analysis to exclude the effects of active psychiatric disorders, we removed five patients with PPPD who showed active psychiatric comorbidities when assessed with the MINI (Tables S1 and S2 in Supplementary Material). Fifteen healthy volunteers were matched for demographic variables, personality traits, anxiety, depression, and motion sickness susceptibility to patients with PPPD (see Table 1).

**Table 1:** Demographic and clinical characteristics in patients with PPPD and healthy controls (HCs).

	HCs (*N* = 15)	Patients with PPPD (*N* = 15)	Group differences
	Mean ± SD	Mean ± SD	*t*, χ^2^, *p* values
Sex	7 M, 8 F	9 M, 6 F	χ^2^ = 0.54, *p* = 0.46
Age	30.13 ± 5.67	33.4 ± 12.45	*t* = −0.92, *p* = 0.36
GAD-7 (state anxiety)	7.47 ± 4.55	8.87 ± 4.81	*t* = −0.82, *p* = 0.42
PHQ-9 (depression)	5.67 ± 5.07	8.67 ± 5.25	*t* = −1.59, *p* = 0.12
**NEO-PI-R personality factors**			
Neuroticism	55.08 ± 9.82	56.24 ± 10.73	*t* = −0.31, *p* = 0.76
Extraversion	53.37 ± 10.23	51.16 ± 7.92	*t* = 0.66, *p* = 0.51
Openness	53.01 ± 10.14	45.30 ± 10.47	*t* = 2.05, *p* = 0.05
Agreeableness	47.53 ± 8.43	43.48 ± 8.42	*t* = 1.32, *p* = 0.20
Conscientiousness	49.64 ± 9.24	49.75 ± 8.73	*t* = −0.03, *p* = 0.97
Motion sickness susceptibility	14.17 ± 11.84	14.25 ± 11.63	*t* = −0.02, *p* = 0.99
Sense of perceived realism	4.9 ± 2.88	5.09 ± 2.59	*t* = −0.17, *p* = 0.86
Dizziness Handicap Inventory	–	34 ± 16.1	–
Duration of disease (months)	–	32.5 ± 34.8	–

*PPPD, persistent postural-perceptual dizziness; GAD-7, Generalized Anxiety Disorder questionnaire; PHQ-9, Patient Health Questionnaire; NEO-PI-R, NEO Personality Inventory, revised*.

### Functional Magnetic Resonance Imaging (fMRI) Task

The fMRI task was delivered *via* an MRI compatible VisualSystem (NordicNeuroLab^3^). This comprised of goggles that have diopter correction and pupil distance adjustment and provides immersion in virtual-reality context while isolating participants from the external environment. AVI videos were displayed *via* PsychoToolbox 3.0.10^4^ running on MATLAB 2012a^5^ at 800 × 600 pixels, 30° × 23° visual angle, 60 frames/s.

**Figure 1:**
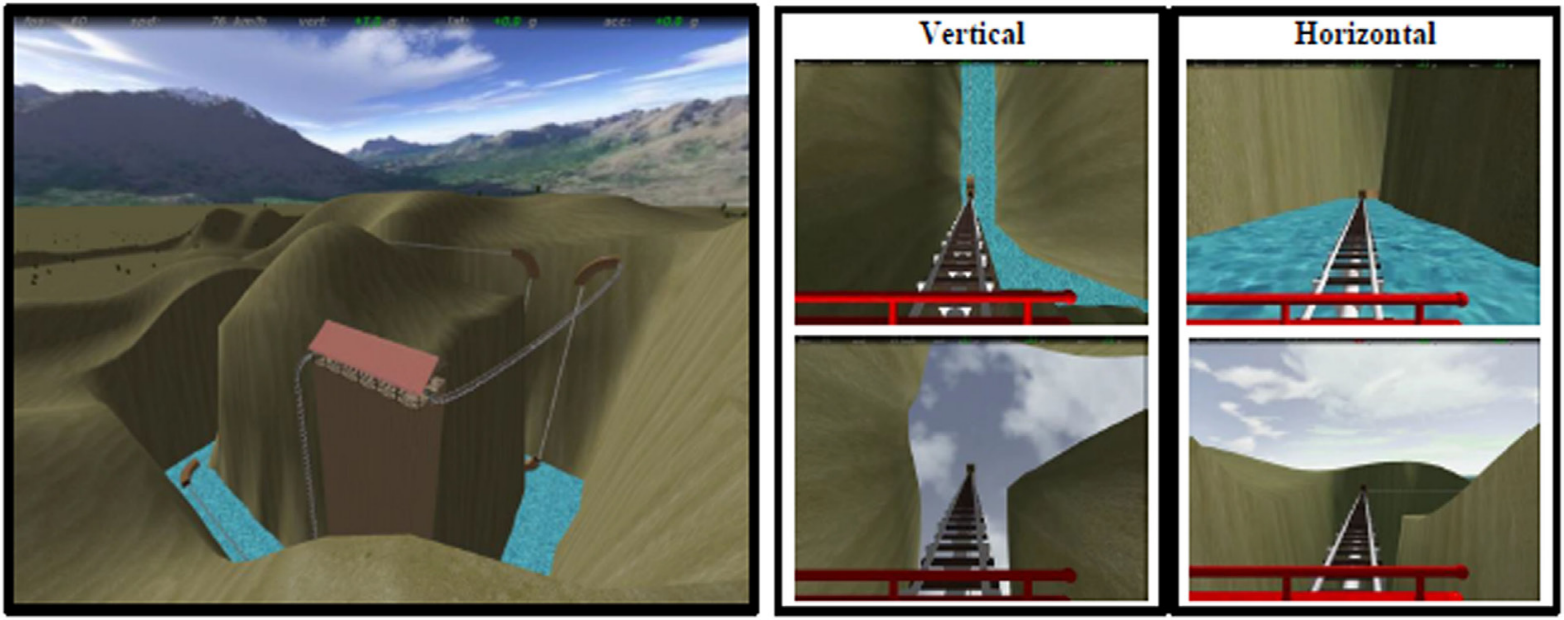
Virtual rollercoaster environment showing panoramic overview (left) and still frames from examples of vertical (central) and horizontal (right) trials.

A detailed description of the task is reported elsewhere (21). Briefly, a ride on a rollercoaster was simulated by showing first person perspective views of animated visual scenes compatible with forward self-motion (Figure 1 and https://www.youtube.com/watch?v=m6QDhipBcqM&feature=youtube for an example of the stimuli). The participants’ view was that of a passenger sitting in the front car and looking straight ahead. A fixation cross was displayed in the center of the scene and corresponded to the focus of expansion during rectilinear motion. The car traveled most of the time in the open air along tracks consisting of vertical and horizontal rectilinear sections, connected by curves. There were also periods during which the car accelerated, decelerated or moved at constant speed (25, 38). To avoid habituation phenomena, we changed the kinematic parameters across trials although these parameters were identical for horizontal and vertical motion trials. The optic flow expanded radially from the central fixation point in both types of trials, and directional cues were provided by the visual scene. The experiment included three sessions in total, each consisting of six movies with a mean duration of 48.25 s (minimum duration: 41.95 s, maximum duration: 56.63 s) presented in a random order. The first frame of each movie was static and lasted 15 s. Details about the kinematics and duration of the vertical and horizontal conditions are reported in Table S3 in Supplementary Material. The total duration of each session was 6 min and 20 s. To ensure that participants paid attention to the stimuli, they were instructed to fixate the cross at the center of the screen and press a button when the color of this fixation cross changed from blue to yellow. The color of the central cross changed six times during the whole experiment.

At the end of the experiment, we asked participants to rate their “sense of presence” (39), that is, the participants’ feeling of being immersed in the virtual environment. More specifically, they had to rate “how strong was the sensation of being on the moving rollercoaster car” on a Likert scale from 0 (“none”) to 10 (“as on a real rollercoaster”) (38).

### Data Acquisition

Neuroimaging data were acquired on a 3 T unit using an 8-channel head coil (Discovery MR-750, General Electric, Milwaukee, WI, USA). Head movements were minimized using foam pads around participants’ heads. Whole-brain fMRI data were acquired through echo planar images (EPIs) sensitive to the blood oxygenation level-dependent (BOLD) contrast (39 axial slices, 3-mm thickness each, repetition time = 2,000 ms, echo time = 30 ms, voxel size 3 mm × 3 mm × 3 mm). Photoplethysmographic signals were collected while participants were in the scanner using a General Electric pulse-oximeter located on the forefinger of the left hand sampling at 10 ms intervals. Finally, movements of the right eye were recorded throughout the task using an EyeTracking Camera integrated into the NordicLab VisualSystem^6^ (resolution of 320 × 240 pixels at 30 frames/s).

### Image Preprocessing

Data were preprocessed with SPM8.^7^ Slice-acquisition delays were corrected using the first slice as reference (ascending order). Low-frequency signal drift was eliminated using a high-pass filter with a cutoff of 128 s. An autoregressive model (AR[1]) was applied to correct for autocorrelations among voxels. EPIs were next realigned to the first scan by rigid body transformations to correct for head movements. None of the participants had head movements >2 mm. Realigned scans were normalized to the standard template in the Montreal Neurological Institute (MNI) space using linear and nonlinear transformations. Finally, images were smoothed with a Gaussian kernel of full width at half maximum of 8 mm (40).

### Heart Rate (HR) Analysis

Heart rate data were available for all subjects, except for three patients with PPPD whose pulse oximetry signals were corrupted. Raw waveforms were analyzed using the open source PhysIO Toolbox (41), which is part of the TAPAS (Translational Algorithms for Psychiatry-Advancing Science) software.^8^ For each session of each participant, inter-beat intervals were extracted from the pulse oximetry waveforms and converted to HR in beats per minute. Results were inspected visually for accuracy, corrected when necessary using the Kubios HRV toolbox (42), and synchronized with the task conditions. The median of HR value was calculated within the duration of each individual trial. Repeated measures analysis of variance (RM-ANOVA) was performed on HR median values with motion direction and motion kinematics as within-subjects factors and group as a between-subject factor. Data preprocessing was performed with custom software in MATLAB. Statistical analyses were performed with SPSS (IBM, Armonk, NY, USA).

### Eye-Movement Analysis

Eye-movements data were used to assess the fidelity of participants’ attention and visual fixation during virtual-reality simulation. Saccadic movements could have indicated attentional biases or lack of ability to fixate the target of interest (the central cross in this experiment). Therefore, we used an in-house script implemented in MATLAB to identify saccades as eye displacements of more than three standard deviations from the baseline signal and lasting more than 100 ms. RM-ANOVA was performed on average number of saccades as described in the previous paragraph. Eye-movement data from five healthy controls (HCs) and three PPPD participants could not be analyzed due to low quality of recordings.

### fMRI Analysis of Regional Responses

For each participant, we constructed a general linear model (GLM) to assess regionally specific effects of task parameters on BOLD activations. Trials were modeled as epochs of variable duration and convolved with the SPM8 hemodynamic response function. First-level GLMs included vertical, horizontal, and static conditions. Curves were modeled separately and not further analyzed. Six realignment parameters were included as covariates of no interest to remove residual motion-related variance. Also, six RETROICOR regressors computed as third-order Fourier expansions (43) and a regressor containing the convolution of HR with the cardiac response function (CRF) (44) were included in the model as covariates of no interest, except in the three patients with corrupted pulse oximetry signals. The CRF regressor was split by vertical, horizontal, and static conditions.

The main contrasts of interest were (1) vertical vs horizontal condition and (2) all motion vs static condition. We performed two-sample *t*-tests (one per each contrast of interest) to explore the differences between HCs and patients with PPPD. For each contrast of interest, we also calculated regression coefficients between brain activity and dizziness handicap (DHI scores) within the PPPD group to test the hypothesis that severity of handicap modulated brain responses in key visuo-vestibular regions.

The analysis of second-level maps was restricted to *a priori* regions of interest including components of the multimodal vestibular cortex, hippocampus, frontal regulatory regions, and visual areas (23). To this end, a single brain anatomical mask was created including the bilateral insulae, Rolandic opercula, inferior frontal opercula, hippocampi, anterior cingulate gyri, calcarine cortices, lingual gyri, and middle occipital gyri extracted *via* the Automated Anatomical Labeling template (45). Moreover, we included the posterior superior temporal gyrus (BA 41 and 42) as derived from the brainnetome atlas (46).

We applied corrections for multiple comparisons as determined by Monte Carlo simulation at the cluster level using family-wise error correction implemented in the SPM RESTplus software package (47). This non-parametric method avoids inflation of false-positive rates occurring with cluster-level corrections (48, 49). It determines the number of contiguous voxels (*k*) needed to survive a cluster-wise corrected significance level. In this study, we performed Monte Carlo simulations running 10,000 iterations within the mask including 34,591 voxels in our regions of interest with an independent voxel threshold of *p* < 0.005 (47). We applied a cluster-wise corrected threshold of *p* < 0.05. We executed one simulation for each comparison between groups (two-sample *t*-tests) and for each correlation analysis. For each simulation, we estimated the inherent smoothness of the data within the mask using the smoothness estimation function of the toolbox entering each statistical T-map as input. The minimum required cluster sizes determined from the Monte Carlo simulations are summarized in Table S4 in Supplementary Material. On average, we used a cluster size of 147.25 ± 2.3 voxels.

## Results

### Behavioral Results

Participants’ age, sex, NEO-PI-R personality scores, MSSQ scores, sense of presence during the rollercoaster simulation, and DHI scores (for patients with PPPD) are summarized in Table 1. Results show that the two groups were matched well for demographics, state anxiety and depression, all five NEO-PI-R personality factors, and motion sickness susceptibility. To further insure that our results were not confounded by an unequal distribution in the range of motion sickness susceptibility (50, 51), we calculated the distribution of participants with low, mild/moderate, and high MSSQ according to normative data (34). 20% of Participants had low susceptibility, 53.3% mild or moderate susceptibility, and 26.7% high susceptibility with no difference between patients with PPPD and HCs. The perceived realism of the task was comparable between the two groups, and no participant reported nausea during the task.

### Heart Rate

Heart rate did not significantly differ as a function of groups [*F*_(1,25)_ = 0.19, *p* = 0.67], motion direction [*F*_(1,25)_ = 0.22, *p* = 0.64], or motion kinematics [*F*_(2,24)_ = 2.6, *p* = 0.10]. Moreover, no significant interactions among these factors were found (all *F*s < 0.42, all *p*s > 0.52).

### Eye-Movement Results

The number of saccadic eye movements did not differ as a function of group (*F* = 0.24, *p* = 0.63), motion direction (*F* = 0.39, *p* = 0.54), or motion kinematics (*F* = 0.002, *p* = 0.99). Moreover, no significant interactions among these factors were found (all *F*s < 1.88, all *p*s > 0.17).

### fMRI Results

#### Vertical vs Horizontal Motion: HCs vs Patients with PPPD

**Figure 2:**
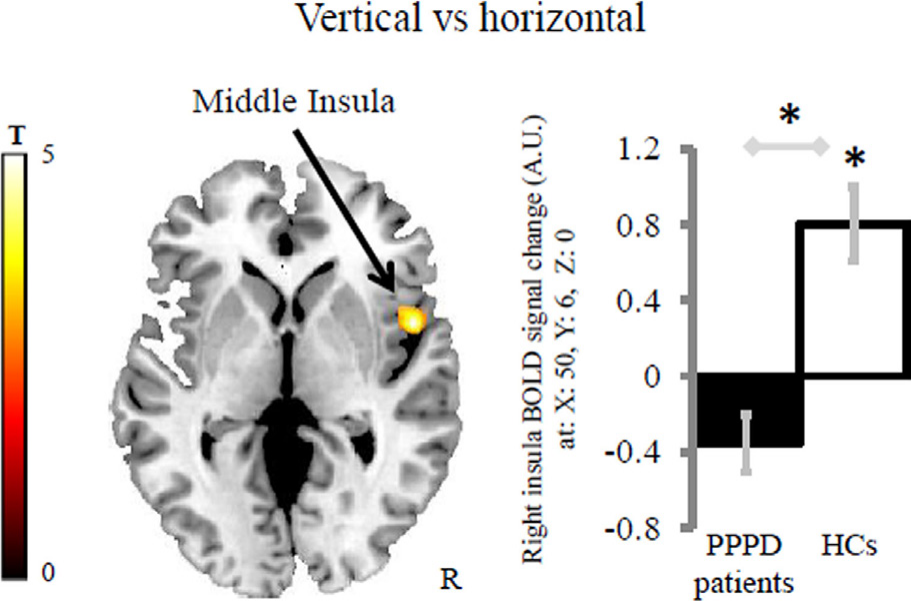
Group differences in the brain response for the contrast vertical vs horizontal. Patients with persistent postural-perceptual dizziness (PPPD) displayed less difference of activity in the right middle insula compared with healthy controls (HCs). Asterisks indicates significant results at cluster-wise corrected threshold of *p* < 0.05 after Monte Carlo simulations within the brain mask including the regions of interest. The coordinates (*x, y, z*) are in the Montreal Neurological Institute space. The color bar represents *t*-statistics. In the bar graph, bars represent the mean BOLD response of each group extracted from the cluster displayed. Error bars represent the Standard Error. BOLD, blood oxygenation level-dependent signal; A.U., arbitrary unit; R, right hemisphere.

Healthy controls displayed greater activation to vertical vs horizontal motion in the third short insular gyrus (right middle insula and anterior to the central insular sulcus) and adjacent Rolandic operculum than patients with PPPD (MNI coordinates: *x* = 50, *y* = 6, *z* = 0; *z*-score = 4.36, *k* = 193 voxels with *k*_min_ = 144 voxels). The within group effect of vertical vs horizontal motion was significant in HCs (MNI coordinates: *x* = 50, *y* = 6, *z* = 0; *z*-score = 4.46, *k* = 248 voxels), but not in patients with PPPD (Figure 2) indicating that the main effect between groups was driven by the response of HCs. This result was not related to psychiatric comorbidity as patients in the PPPD group with and without active psychiatric disorders showed similar responses (see Figure S1A in Supplementary Material).

No regions survived the horizontal vs vertical contrast when comparing PPPD patients vs HCs. We also tested the effect of the conditions independently of groups. These findings are reported in Supplementary Material.

#### Vertical vs Horizontal Motion: Correlation of Brain Activity with Severity of Dizziness Handicap in Patients with PPPD

**Figure 3:**
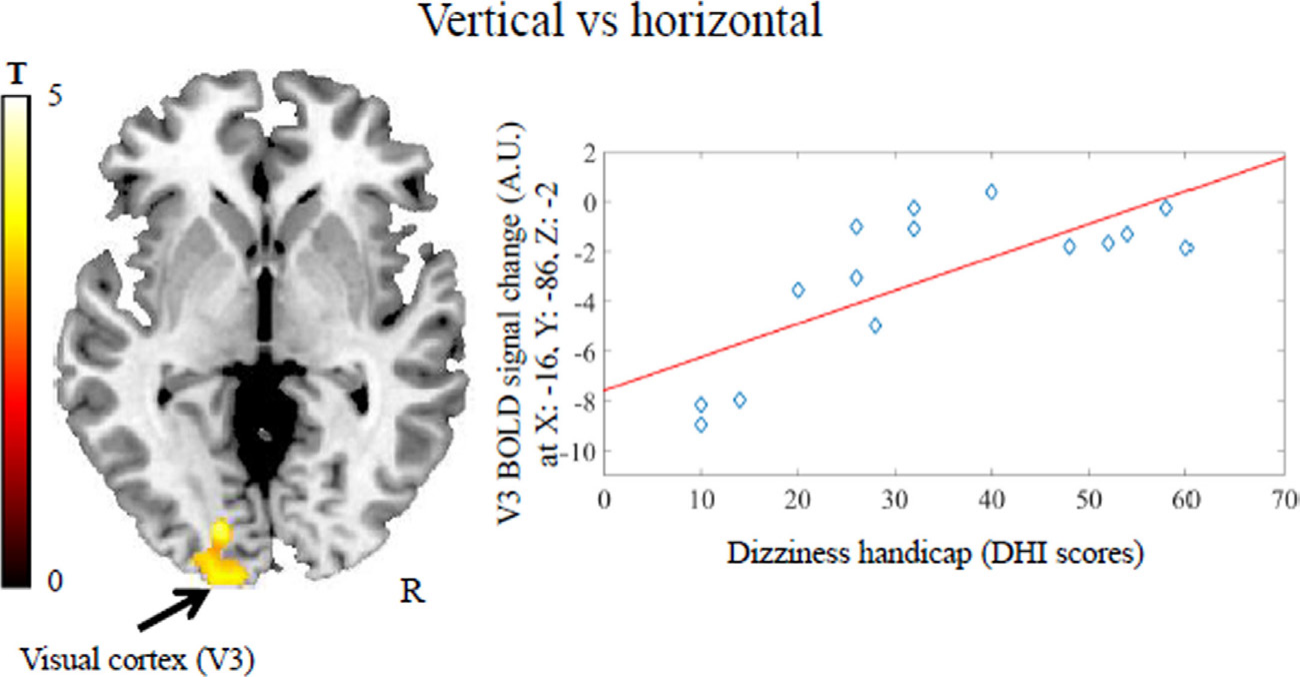
Positive association between dizziness handicap [i.e., scores on the Dizziness Handicap Inventory (DHI)] and visual cortex activity (V3) in patients with persistent postural-perceptual dizziness for the contrast vertical vs horizontal motion. This result survived a cluster-wise corrected threshold of *p* < 0.05 after Monte Carlo simulations within the brain mask including the regions of interest. The coordinates (*x, y, z*) are in the Montreal Neurological Institute space. Each dot represents individual mean BOLD responses within the displayed cluster; red line represents the regression line. The color bar represents *t*-statistics. BOLD, blood oxygenation level-dependent signal; A.U., arbitrary unit; R, right hemisphere.

Activation during the vertical vs horizontal comparison correlated positively with dizziness handicap in a visual cortical area comprising V1, V2, and V3 bilaterally (MNI coordinates: *x* = −16, *y* = −86, *z* = −2; *z*-score = 3.28, *k* = 181 voxels and MNI coordinates: *x* = 14, *y* = −84, *z* = −4, *z*-score = 3.50, *k* = 168 voxels; *k*_min_ = 151 voxels) (Figure 3). This result was not due to the presence of psychiatric comorbidity in five of the patients with PPPD (see Figure S1B in Supplementary Material). No regions negatively correlated with dizziness handicap.

#### All Motion vs Static Conditions: HCs vs Patients with PPPD

No significant clusters were found that exceeded the threshold of 148 contiguous voxels when comparing HCs vs patients with PPPD for the contrast of all motion vs static conditions. Furthermore, no regions survived the opposite comparison. There were no significant correlations between brain responses to the all motion vs static contrast and severity of dizziness handicap in patients with PPPD. The effect of conditions independently of group is reported in Supplementary Material.

## Discussion

In this study, we investigated for the first time brain responses of patients with PPPD to a visual motion stimulus representing rollercoaster rides.

Patients with PPPD were matched with HCs in terms of age, gender, personality scores, as well as state and trait anxiety, and motion sickness susceptibility.

To control for the effect of heart pulsatility on brain activity, we included cardiac regressors as covariates of no interest in the fMRI analysis. We also directly assessed HR and eye-movement differences between conditions and groups and did not find any significant effects.

In contrast to HCs who showed an increase of activity in the middle insula when comparing vertical vs horizontal conditions, we found that patients with PPPD did not show such a difference in activity. For the same comparison, we also identified increased activity in visual cortical areas only in patients with PPPD as a function of the severity of their dizziness handicap. Taken together, these results indicate that the middle insular response was more closely associated with spatial motion encoding than arousal effects in HCs, and this encoding was disrupted in patients with PPPD.

The posterior-middle insular cortex has been found to display significantly increased activity in response to vestibular stimulation (52, 53). This part of the insula is considered a component of the multimodal vestibular cortex (53). In particular, it has been shown to encode *a priori* knowledge about visual effects of gravity during self-motion along the vertical relative to the horizontal direction (25). This *a priori* knowledge is used to accurately time body movements in space and successfully regulate the body interaction with the environment (26, 27, 38, 52, 54, 55). Vestibular inputs affect this process as demonstrated in a behavioral study that used sound-evoked vestibular stimulation to produce a conflicting vestibular input during the visual rollercoaster simulation (56). In absence of perturbing vestibular inputs, participants were able to anticipate the effects of gravity when calculating their arrival time at a target during the vertical relative to the horizontal condition (38, 56). The artificial vestibular signal disrupted this ability (56). Moreover, patients with infarcts in a perisylvian region adjacent to the posterior insula had less ability to discriminate visual motion with natural gravitational acceleration (28).

In a previous fMRI study, we found that the response of the posterior-middle insular region to sound-evoked vestibular stimuli was decreased in patients with PPPD relative to HCs (23). Together with the results of this study, these findings suggest a general downregulation of activity or hyporesponsivity of this region to motion stimulation across vestibular and visual modalities. This reduced activation in patients with PPPD may affect the predictive mechanisms normally used to regulate self-motion through *a priori* knowledge about gravity. It is possible that this change in cortical activity is related to postural symptoms and alterations in postural control observed recently in patients with PPPD (8) and previously reported in patients with PPV (11, 12) and CSD (13), but this hypothesis awaits investigation in future studies.

Symptoms of chronic non-vertiginous dizziness and unsteadiness similar to that experienced by patients with PPPD have been related to increased visual dependence following VN (14, 15). Visual dependence is thought to make patients more vulnerable to visually induced dizziness (i.e., unsteadiness or dizziness triggered by exposure to complex or moving visual stimuli), a key symptom of PPPD, by reweighting the processing of vestibular, visual and somatosensory space-motion information to favor visual inputs (1, 2, 14). Our finding of increased visual cortical activity in patients with PPPD that correlated with severity of dizziness handicap is in keeping with this concept. Other studies have reported alterations in visual cortical connectivity in patients with visually induced dizziness (22) and PPPD (23). In our previous work with the same group of patients with PPPD who participated in this set of experiments (23), we found more negative connectivity between anterior insula and visual occipital areas in patients with PPPD compared with HCs in response to sound-evoked vestibular stimulation (23). These results suggest that complex alterations in connectivity between primary visual cortex, visual association areas, and other regions of the brain that process and regulate responses to multimodality space-motion information may underlie visually induced dizziness and the responses of patients with PPPD to visual motion and visual orientation cues. Increased visual cortical activity was previously associated with top-down attentional effects (57). The potential association of visually related symptoms, visual dependence, increased attention, and activity and connectivity patterns in visual cortical regions in patients with PPPD requires additional research.

Our novel findings of abnormal responses in vestibular and visual regions of the brain in patients with PPPD and their relationship to the severity of dizziness handicap are intriguing and promising. Specifically, we detected functional alterations in vestibular and visual systems of the brain that may provide a mechanistic explanation for the problems with postural control and greater sensitivity to visual stimuli experienced by patients with PPPD, although future studies are needed to better refine the pathophysiology of this disorder and the specificity and generalizability of the current results. For example, it is not known if patients who developed PPPD triggered by illnesses other than peripheral vestibular episodes (e.g., psychiatric disorders, metabolic dysfunctions, dysregulation of the autonomic system) show similar brain abnormalities compared with HCs.

A possible limitation of this study was the relatively small sample of participants. However, this limitation was offset by the strengths of a well characterized and uniform patient cohort and the close match of patients with HCs, which allowed us to control for psychological factors such as personality traits and affective state that are known to complicate the clinical presentation of PPPD and confound investigations of this type.

## Ethics Statement

All participants gave written informed consent to participate in the study, which was approved by the University of Catanzaro Research Ethics Committee, according to the Helsinki declaration (http://www.wma.net/en/30publications/10policies/b3/).

## Author Contributions

RR significantly contributed to data acquisition and analysis and drafted the manuscript. LP contributed to the study concept and design and provided critical revision of the manuscript. NT gave a contribution in the heart-rate data analysis and critically revised the manuscript. SN gave a contribution in the eye-movement analysis. GC and CP evaluated clinical signs and symptoms in PPPD patients. FL critically revised the manuscript. JS helped with design definition and critically revised the manuscript. II was responsible for the study concept and design, significantly contributed to the interpretation of data for the work, and drafted the manuscript. All the authors reviewed the content of the manuscript and approved its final version for submission.

## Conflict of Interest Statement

The authors declare that the research was conducted in the absence of any commercial or financial relationships that could be construed as a potential conflict of interest.

## Footnotes

^1^https://www.wma.net/policies-post/wma-declaration-of-helsinki-ethical-principles-for-medical-research-involving-human-subjects/.

^2^http://www.who.int/classifications/icd/en/.

^3^http://www.nordicneurolab.com/Products_and_Solutions/fMRI_Hardware/VisualSystem.aspx.

^4^https://github.com/Psychtoolbox-3/Psychtoolbox-3.

^5^www.matworks.com.

^6^http://www.nordicneurolab.com/products/VisualSystem.html.

^7^http://www.fil.ion.ucl.ac.uk/spm/.

^8^www.translationalneuromodeling.org/tapas/.
