# Supplementary material for: Altered Insular and Occipital Responses to Simulated Vertical Self-Motion in Patients with Persistent Postural-Perceptual Dizziness
Source: Front Neurol. 2017 Oct 17;8:529. doi: 10.3389/fneur.2017.00529 (PMC5650964; doi:10.3389/fneur.2017.00529)
Supplement: Supplementary file 1 [file data_sheet_1.docx]

**Supplementary Results**

**All visual motion versus rest**

**Healthy controls and patients**. The comparison of all visual motions with the static condition elicited significant activation in a large visual area and in the hippocampus (maximum of the cluster in the mid occipital gyrus, MNI coordinates x: 28, y: -88, z: 20, z-score>10, k = 12513), and in the anterior cingulum (MNI coordinates x:4, y: 52, z:24, z-score=3.37, k=153). It also elicited significant activation in the posterior portion of the Rolandic operculum (MNI coordinates x:-40, y:-26, z:22, z-score=3.49; k=122), previously defined as PIVC or PIC (Riccelli et al., 2016). On the contrary visual motion deactivated a region encompassing the Rolandic operculum and the whole insula (MNI coordinates x:56, y:8, z:2, z-score=4.28; xyz = 44 -14 -4, z-score=3.94, k=550), and the subgenual cingulate cortex (MNI coordinates x:-8, y:32, z: -4, z-score=3.86, k=214). As all analyses reported in the main text, this analysis was thresholded using a p-value of 0.005 and a minimum cluster size of 148 voxels as calculated via Monte Carlo simulation within the regions of interest.

**Main effect of vertical vs horizontal motion**

**Healthy controls and patients.** The comparison of all vertical with horizontal motion conditions across groups of participants elicited significant activation in visual areas both in vertical bigger than horizontal (calcarine region, MNI coordinates x:6, y:-72, z:10, z-score = 5.19, k = 1822; mid occipital cortex, MNI coordinates x: -52, y:-70, z:2, z-score = 4.38, k = 450, MNI coordinates x:48, y:-70, z:6,, z-score = 3.75, k = 149) and horizontal bigger than vertical (lingual gyrus, MNI coordinates x:-14, y:-90, z:-8, z-score = 6.75, k = 2191; -26 -48 -8, z-score = 6.27, k = 149; calcarine region, MNI coordinates x:18, y:-96, z:4,, z-score = 5.49, k = 601). As all analyses reported in the main text, this analysis was thresholded using a p-value of 0.005 and a minimum cluster size of 144 voxels as calculated via Monte Carlo simulation within the regions of interest.

Table S1

|  |  |  |  |  |
| --- | --- | --- | --- | --- |
| Subjects Identifier | **Generalized Anxiety** | **Panic attack** | **Agoraphobia** | **PTSD** |
| PPPD #1 | x |  |  | x |
| PPPD #7 |  | x |  |  |
| PPPD #9 |  | x |  |  |
| PPPD #10 |  | x | x |  |
| PPPD #14 |  | x |  |  |

List of the active psychiatric conditions in the group of patients with persistent postural-perceptual dizziness (PPPD) and healthy controls. *PTSD, Post-Traumatic Stress Disorder.*

Table S2

|  | Healthy controls (N=15) | Patients with PPPD (N=10) | Group differences |
| --- | --- | --- | --- |
|  | Mean±SD | Mean±SD | t, χ^2^, p values |
| Sex | 7 M, 8 F | 7 M, 3 F | χ^2^=1.09, p=0.29 |
| Age | 30.13±5.67 | 33.3±13.65 | t=-0.69, p=0.50 |
| GAD7 (state-anxiety) | 7.47±4.55 | 8.1±4.98 | t=-0.32, p=0.75 |
| PHQ9 (depression) | 5.67±5.07 | 7.9±5.61 | t=-1.01, p=0.32 |
| NEO-PI-R Personality Factors |  |  |  |
| Neuroticism | 55.08±9.82 | 53.96±9.08 | t=0.29, p=0.77 |
| Extraversion | 53.37±10.23 | 51.41±7.92 | t=0.53, p=0.60 |
| Openness | 53.01±10.14 | 48.43±7.68 | t=1.27, p=0.21 |
| Agreeableness | 47.53±8.43 | 41.52±9.38 | t=1.63, p=0.12 |
| Conscientiousness | 49.64±9.24 | 46.70±7.16 | t=0.89, p=0.38 |
| Motion Sickness Susceptibility | 14.17±11.84 | 13.23±13.60 | t=0.18, p=0.86 |
| Sense of Perceived realism | 4.9±2.88 | 4.57±1.98 | t=0.31, p=0.76 |
| Dizziness Handicap Inventory (DHI) | - | 30.8±17.2 | - |
| Duration of disease (months) | - | 41.7±40.8 | - |

Demographic and clinical characteristics in patients with PPPD and healthy controls after removing the 5 patients with PPPD who had active psychiatric comorbidities as listed in Table S1.

| Table S3 |  |  |  |
| --- | --- | --- | --- |
|  | **Mean (s)** | **Standard Deviation (s)** | **Range (s) [min-max]** |
| Horizontal motion accelerated | 4.02 | 0.62 | [3.22-4.77] |
| Horizontal motion decelerated | 4.11 | 0.82 | [3.07-4.87] |
| Horizontal motion, constant speed | 4.02 | 0.72 | [3.16-5] |
| Vertical motion, accelerated down | 4.09 | 0.61 | [3.37-4.73] |
| Vertical motion, decelerated up | 3.92 | 0.74 | [3.05-4.73] |
| Vertical motion, constant speed up | 3.94 | 0.49 | [3.32-4.63] |
| Vertical motion, constant speed down | 4.07 | 0.59 | [3.38-4.72] |
| Static condition | 15.00 | 0.003 | [15.00-15.00] |
|  |  |  |  |

Details about the duration of each condition of interest included in the rollercoaster simulation.

| Table S4 |  |  |
| --- | --- | --- |
| Comparison of interest | **k_min_** | |
| Vertical vs horizontal motion (two sample t-test) | 144 | |
| All motion trial vs static condition (two-sample t-test) | 148 | |
| Vertical vs horizontal motion, correlation analysis with DHI total score | 151 | |
| All rectilinear motion versus static condition, correlation analysis with DHI total score | 146 | |

Minumum cluster size (number of contiguous voxels) calculated for each comparison through Monte Carlo simulation. The individual voxel threshold was set to p=0.005 and 10000 simulations were computed. For each statistical map, the inherent smoothness was also calculated.


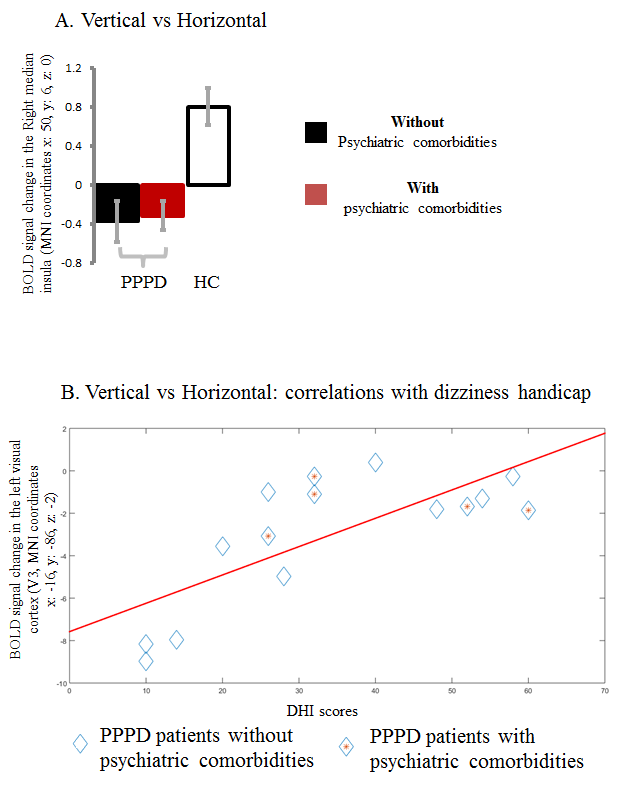


Fig. S1: Main finding for the group differences and correlation analysis splitting the PPPD group into two subgroups (without and with psychiatric comorbidities). Red points and bars are patients with PPPD and psychiatric comorbidities, empty diamonds and black bars are patients with PPPD but no psychiatric comorbidities. Bars are the mean BOLD response extracted from the cluster in the middle insula displayed in Fig. 3. Diamonds represents the individual mean BOLD responses within the cluster displayed in Fig. 4. The coordinates (X, Y, Z) are in the Montreal Neurological Institute (MNI)
